# Healthcare Professional Perspectives on the Use of Remote Patient-Monitoring Platforms during the COVID-19 Pandemic: A Cross-Sectional Study

**DOI:** 10.3390/jpm12040529

**Published:** 2022-03-25

**Authors:** Khayreddine Bouabida, Kathy Malas, Annie Talbot, Marie-Ève Desrosiers, Frédéric Lavoie, Bertrand Lebouché, Niloofar Taghizadeh, Louise Normandin, Cécile Vialaron, Olivier Fortin, David Lessard, Marie-Pascale Pomey

**Affiliations:** 1 Research Center of the Hospital Center of the University of Montreal (CRCHUM), Montreal, QC H2X 0A9, Canada; khayreddine.bouabida@umontreal.ca (K.B.); louise.normandin.chum@ssss.gouv.qc.ca (L.N.); cecile.vialaron.chum@ssss.gouv.qc.ca (C.V.); olivier.fortin03@gmail.com (O.F.); 2 Department of Health Management, Evaluation, and Policy, School of Public Health, University of Montreal, Montreal, QC H3N 1X9, Canada; 3 Hospital Center of the University of Montreal (CHUM), Montreal, QC H2X 0C1, Canada; kathy.malas.chum@ssss.gouv.qc.ca (K.M.); annie.talbot.med@ssss.gouv.qc.ca (A.T.); marie-eve.desrosiers.chum@ssss.gouv.qc.ca (M.-È.D.); frederic.lavoie.md@gmail.com (F.L.); 4 Canadian Institutes of Health Research Strategy for Patient-Oriented Research Mentorship Chair in Innovative Clinical Trials in Human Immunodeficiency Virus (HIV), Montreal, QC K1A 0W9, Canada; bertrand.lebouche@mcgill.ca; 5 Centre for Outcomes Research and Evaluation, Health Centre Research Institute, McGill University, Montreal, QC H4A 3S9, Canada; david.lessard2@mail.mcgill.ca; 6 STATCure Consulting Services Inc., Calgary, AB T3K2A8, Canada; niloofar.a.taghizadeh@gmail.com; 7 Centre of Excellence for Partnership with Patients and the Public, Montreal, QC H2X 0A9, Canada

**Keywords:** COVID-19, health professionals, telehealth, remote monitoring, survey

## Abstract

The COVID-19 pandemic created an urgent need to act to reduce the spread of the virus and alleviate congestion in healthcare services, protect health professionals, and help them maintain satisfactory quality and safety of care. Remote monitoring platforms (RPM) emerged as potential solutions. In this study, we evaluate, from health professionals’ perspectives, the capacity and contribution of two different digital platforms to maintain quality, safety, and patient engagement in care. A cross-sectional study was conducted using a survey in which a total of 491 health professionals participated. The results show that, in general, user perceptions of the quality and safety of care provided through the platforms were positive. The ease of access to health professionals’ services in general and shorter waiting times for patients were the two main features that were highly appreciated by most participants. However, some problems were encountered during the use of these two platforms, such as a lack of training and/or direct support for users. To improve the two platforms and maximize their use, the areas for improvement and the issues identified should be addressed as part of a collaborative process involving health professionals and patients as well as health system leaders, decision-makers, and digital platform providers.

## 1. Introduction

On 11 March 2020, COVID-19 was declared a pandemic by the World Health Organization, one that was rapidly expanding globally [1]. As of 12 October 2021, it is estimated that COVID-19 had resulted in 237,655,302 confirmed cases and 4,846,981 deaths in 183 countries [1]. In Canada, 1,667,575 cases had been reported, and 28,289 deaths had been attributed to COVID-19 [2].

Quebec has been one of Canada’s most impacted provinces, with 417,188 cases and 11,429 deaths as at the same date [3]. At the time COVID-19 was declared a pandemic on 11 March 2020, in Quebec, Canada and many other places and countries around the world, various preventive measures were implemented by the local authorities to contain the spread of the virus, including closures of schools and public places, and curfews as well as the quarantining of cities [4]. These measures created challenges for health care delivery, with negative impacts on access to care, performance, health outcomes, and quality of care, and the situation exposed all healthcare stakeholders to risks of isolation, anxiety, and depression [5,6,7,8,9,10].

By spring 2020, no vaccine or effective treatment had been developed. With a growing number of confirmed cases and deaths, the COVID-19 pandemic posed a huge challenge to Quebec’s healthcare system [7,8]. The use of health resources, including personnel, beds, and facilities, was at maximum capacity, and health care workers were under great pressure and experiencing significant distress, especially physicians and nurses [5,6,11,12,13,14].

Health care workers worried about contracting the infection themselves or passing it on to patients or to their loved ones, in addition to their concerns about maintaining satisfactory quality and safety of care, and their performance was affected due to the increased workload and changes to protocols [5,6,9,10,11,12,13]. One lesson that was promptly learned from the COVID-19 pandemic was the need to optimize the provision of health care outside of traditional settings—and potentially over longer periods of time—and to adapt it to unpredictable contexts [14].

In order to adapt to the particular nature of COVID-19, support the health care workers as they strived to maintain good quality and safety of care, and help reduce transmission of the virus, a program named the “Techno-COVID Partnership” (TCP) was implemented at Centre Hospitalier de l’Université de Montréal (CHUM), a university-affiliated hospital in Montreal, Québec, Canada [15]. Among other services, this program included two innovative technological platforms that had been created before the pandemic for online consultations and remote patient monitoring; however, the pandemic required that they be developed and adapted to remotely monitor patients with COVID-19.

The first platform in this program is TELECARE (henceforth, Telecare-Covid), and the second platform is REACTS Teleconsultation (henceforth, REACTS-Teleconsultation). Telecare-Covid is a telementoring platform for providing clinical follow-up of outpatients following a hospital visit or discharge. Clinical nurses working at the CHUM coordination center follow the non-continuous monitoring data of patients, and patients have 24/7 access to a nurse to assess their COVID-19 or other symptoms.

Patients can also discuss their clinical symptoms directly with a nurse, outside of Telecare-Covid, who processes and assesses the clinical information by phone or in a virtual evaluation [15]. REACTS-Teleconsultation is a collaborative telehealth platform on which both patients and health professionals meet remotely instead of in a classic in-person appointments [16]. It is a digital platform on which health professionals care for patients through live video consultations, thus, serving as a virtual clinic.

Health care professionals can arrange appointments with patients, perform remote examinations and virtual consultations, recommend treatment, and offer follow-up care, all over a secure, high-quality video and audio connection. REACTS-Teleconsultation can be accessed directly on a web page through a link that is provided to the patient when an appointment is scheduled or through a REACTS app that is downloadable on connected devices, such as smartphones and tablets. In sum, the two platforms can be used to cover remotely the entire trajectory of COVID 19 patients’ post-hospitalization period and/or post visits.

Although these two platforms operate in distinctly different ways, they have nevertheless been developed and adapted to achieve the same goals, which are to provide: (1) a safer return home for patients who are medically stabilized but at risk of decompensation, by guaranteeing regular clinical follow-up and non-continuous remote monitoring for a minimum of 14 days; (2) emotional support to reduce isolation and anxiety in patients by providing a connection to clinical teams; (3) a medical safety net to reduce the risk of SARS-CoV-2 infections within care services; (4) improved workflows and reduced congestion in care services that have been exacerbated by the pandemic, through better control of unnecessary visits to care services and facilities; and (5) eventually, continued care of good quality and safety [15,16].

In this context, we developed this cross-sectional study to evaluate and explore care service delivery and the use of these two technological platforms from users’ points of view, i.e., health care professionals, during the COVID-19 pandemic.

The objectives of this study were to evaluate and explore health professionals’ perceptions of: (1) the quality and safety of care provided through the two technological platforms during the COVID-19 pandemic; (2) patient engagement and partnership in care when each of these technological platforms is used; and (3) the role and relevance of the two technological platforms in terms of usefulness, advantages and problems, and limitations; throughout the patient trajectory (from diagnosis to return home after hospitalization).

## 2. Materials and Methods

This study received ethical approval from the research ethics committee of the research center of Université de Montréal (CRCHUM) (CER-CHUM: 20.040, 23 April 2020).

### 2.1. Study Design

A cross-sectional quantitative study was conducted using a survey [17,18,19,20,21].

### 2.2. Study Population and Recruitment of Participants

The study population included health professionals from CHUM who experienced and used REACTS-Teleconsultation for patients hospitalized and/or referred to Telecare-Covid for patients who need telehealth before or after hospitalization during the first wave of the COVID-19 pandemic (April, May, and June 2020).

We classified each of the health professionals into one of four different categories: (1) physicians working in hot COVID care units; (2) nurses from several care units including hot or cold COVID care units; (3) non-physician/non-nurse health care professionals, such as those working in occupational therapy, the respiratory therapy operating room, clinical nutrition, speech therapy, physiotherapy, kinesiology, radiology, radiation oncology, or nuclear medicine who worked in hot or cold COVID care units; and (4) a category that we call “Other,” which includes health professionals in psychology, social services, and spiritual therapy, and volunteers working in hot or cold COVID care units.

A cold zone/unit is an area of patients or residents with no infections. No special infection prevention precautions are required other than routine hand sanitizing. A hot zone/unit is an area of patients or residents who have a COVID-19 related infection. Infection prevention measures with personal protective equipment are required.

We structured the study population in this manner according to the health professionals’ responsibilities and the nature of their interactions with patients and the platforms, i.e., physicians’ interactions with patients on the platform differ from those of nurses, and the interactions of health professionals in physical and respiratory therapy and clinical nutrition are different from those of health professionals in psychology and social services. Therefore, their perception of the added value of the platform and their experience might differ as well. An email with a link to the questionnaire was sent to all such health care professionals asking them to participate in the study by completing the questionnaire. No exclusion criteria were applied.

### 2.3. Survey

To achieve the study’s objectives and build the survey, we adapted three validated questionnaires to the COVID-19 context in order to evaluate health care professionals’ perceptions of the following dimensions [22,23,24]:

(1) Quality and safety of care (access, safety, relevance, timeliness, etc.) [22].
(2) Patient engagement and partnership with physicians and health care professionals (confidence/trust, autonomy, decision making, information sharing, personal context, empathy, and expertise) as per the CADICEE tool [23].
(3) Perceptions of the technology [24].
(4) The sociodemographic characteristics of the health professionals who used the two platforms [24].

The questionnaire was adapted in two ways. First, the questions were linked to the COVID-19 context. The instructions for the original questionnaire suggest, when administering the items, to start the question by linking it to the disease or the clinical problem for which the health professional or patient is using the technological platform. For example, in the context of the COVID-19 health crisis and for a health care professional, the question could be “Is the REACTS/Telecare platform a good response to my needs?”.

The second way that the questionnaire was adapted involved collecting demographic information: age, gender, profession, and years of experience. We originally wanted to collect demographic data on socioeconomic status and ethnic background, but in the testing phase to determine the acceptability of the survey prior to its official administration, we found that 80% of respondents left the spaces blank for those demographic questions, so we decided not to include them.

The questionnaire was finalized and administered to the participants. It included 21 questions grouped into four sections. Among them, 14 were rated on a 5-point Likert scale (1—completely disagree to 5—completely agree), four were open questions, and three were multiple-choice questions (Table 1).

### 2.4. Data Collection

The data were collected online from July to September 2020. After the participants had given their consent, they were asked to complete the questionnaire. Then the data collected were entered and recorded in CRCHUM’s secure “REDCap©” software (REDCap, Nashville, TN, USA) [25], which was designed specifically for surveys and quantitative data collection and processing.

### 2.5. Statistical Analysis

Survey results were summarized, graphed, and reported using IBM^®^ SPSS^®^ Statistics (IBM^®^ SPSS, Stanford, CA, USA) [26] and Stata (Stata group, College Station, TX, USA) [27]. Quantitative variables are expressed as the mean ± a standard deviation (SD) for normally distributed data and as a median (interquartile range (IQR)) for non-normally distributed data. Categorical data are expressed as numbers (percentages). Fisher’s exact test was used to compare perceptions of the performance, safety, and quality between the various groups of health care professionals. Finally, a statistic for the demographic data was suppressed if the number of actual records used in the calculation was less than 5, in keeping with Statistics Canada guidelines [28].

## 3. Results

### 3.1. Characteristics of Participants

A total of 1545 health professionals were asked to participate in the study. Among those, 491 (31.8%) responded, and their data were included in the analyses. Of this group, 294 (59.9%) were non-physician/non-nurse health care professionals, 81 (16.5%) were nurses, 76 (15.5%) were “other” health care professionals, and 40 (8.2%) were physicians.

The characteristics of the participants are shown in Table 2. A total of 128 participants used REACTS-Teleconsultation, and 34 participants used Telecare-Covid (Table 2). Females and individuals aged 35–44 years old comprised the largest groups of participants by gender and age who evaluated their perceptions of added value for both of the platforms. The median length and standard deviation (SD) of practice at CHUM were 10 (13.3) years and 10 (13.0) years among the participants for REACTS-Teleconsultation and Telecare-Covid, respectively.

Participants who used REACTS-Teleconsultation were more often non-physician/non-nurse health care professionals (66.9%), followed by nurses (6.3%), other (15.0%), and physicians (11.8%). However, the proportion of physicians and non-physician/non-nurse health care professionals among REACTS-Teleconsultation users was higher than among non-REACTS-Teleconsultation users (*p*-value: <0001). The majority of participants who evaluated the Telecare-Covid platform were physicians (47.1%) (Table 2).

Only a portion (155/491 × 100 = 31.6%) of our entire cohort completed the survey on REACTS-Teleconsultation and Telecare-Covid. Many of the respondents who used these two technologies also used one or more other platforms and/or services (multiple options were allowed). This study, embedded in a larger project called the “Techno-COVID Partnership” (TCP), which includes several technologies offered during the first wave of covid19, is focused on the REACTS-Teleconsultation and Telecare-Covid platforms.

### 3.2. Perceptions of the Performance, Quality, and Safety of Care

**Figure 1:**
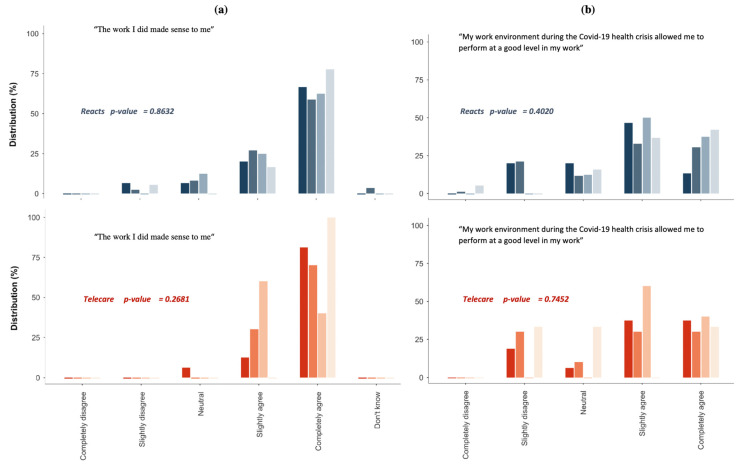

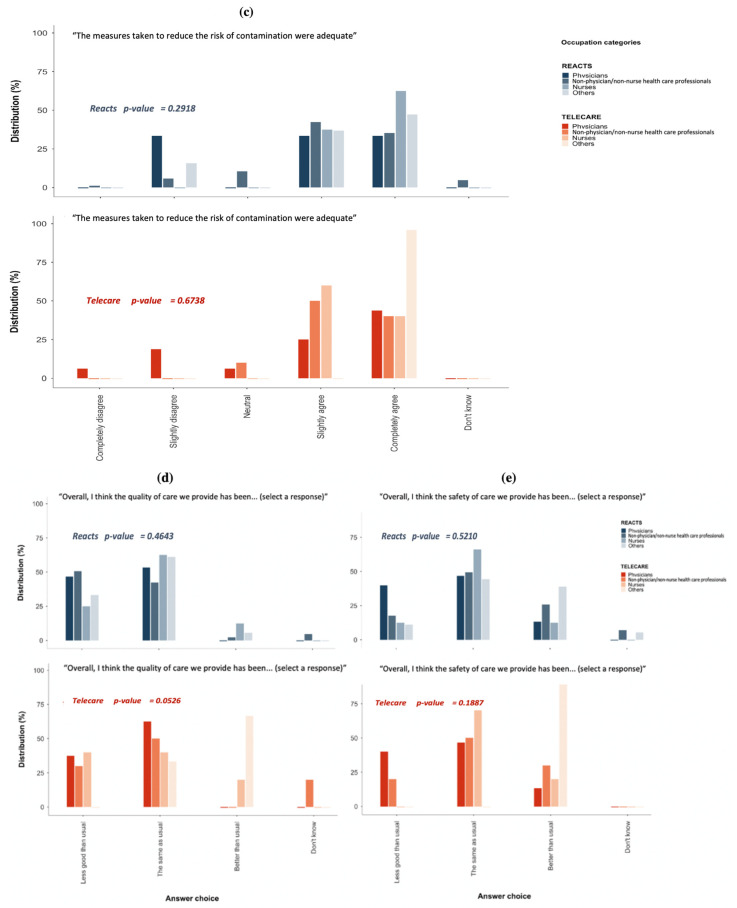
Perceptions of the performance, quality, and safety of care. Figure 1 (**a**). Respondents’ perception of sense of work provided on REACTS platform vs. TELECARE Platform; (**b**). Respondents’ perception of work environment and performance provided on REACTS platform vs. TELECARE Platform Figure 1; (**c**). Respondents’ perception of reducing contamination measures provided on REACTS platform vs. TELECARE Platform; (**d**). Respondents’ perception of quality of care provided on REACTS platform vs. TELECARE Platform; (**e**). Respondents’ perception of safety of care provided on REACTS platform vs. TELECARE Platform.

Figure 1 shows the perceptions of performance, quality, and safety of care as reported by REACTS-Teleconsultation and Telecare-Covid users. Most participants reported that their work during the COVID-19 pandemic made sense to them (Figure 1a), and that the pandemic had a little impact on their performance (Figure 1b). In addition, the majority of the participants believed that the measures taken to reduce the risk of contamination were adequate (Figure 1c).

**Figure 2:**
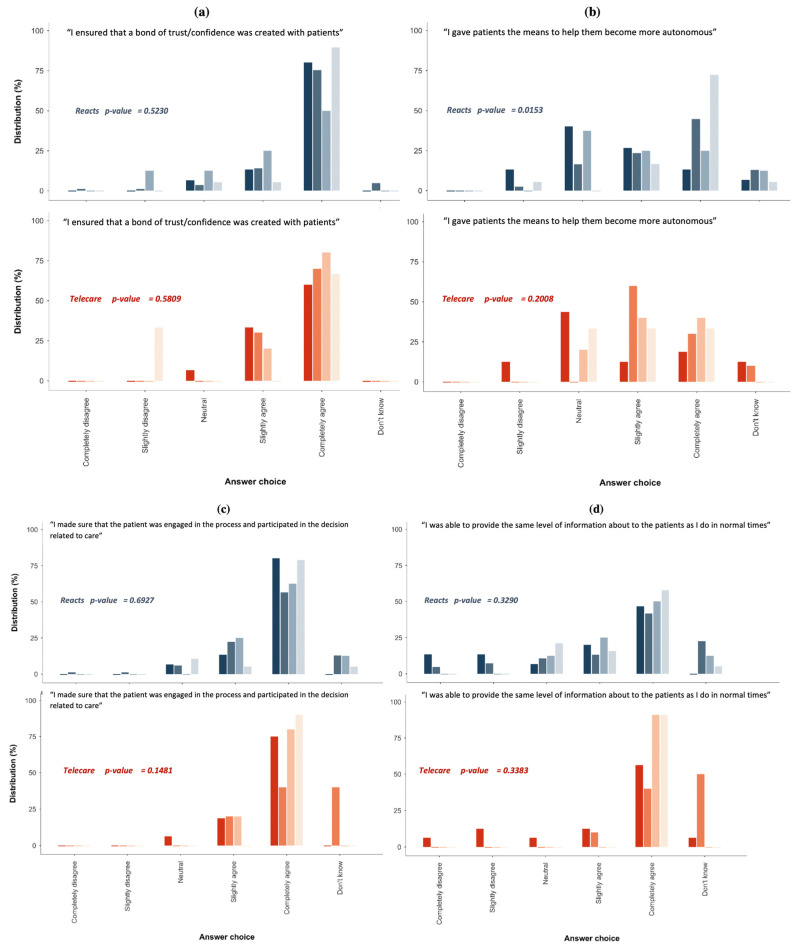

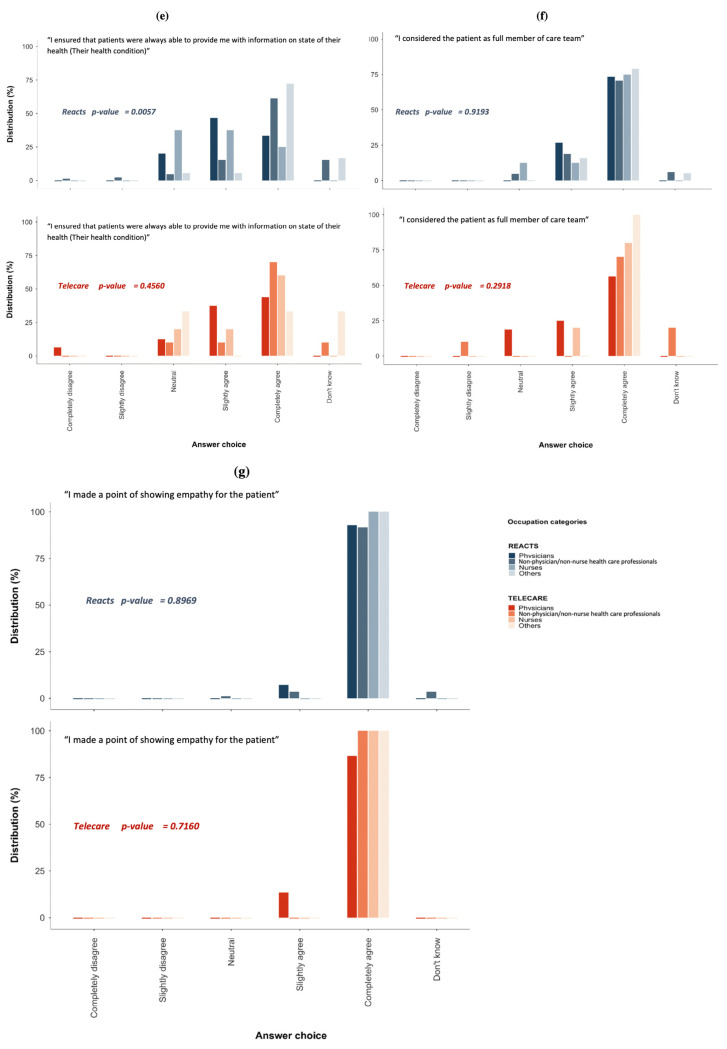
Perceptions of patient engagement in care and the relationship with the care team. Figure 2 (**a**). Respondents’ perception of the bond of trust and confidence created with patients provided on REACTS platform vs. TELECARE Platform; (**b**). Respondents’ perception of the means given to help patients to become autonomous provided on REACTS platform vs. TELECARE Platform; (**c**). Respondents’ perception of the patient engagement in the decision-making process of care provided on REACTS platform vs. TELECARE Platform; (**d**). Respondents’ perception of the level of information to patients provided on REACTS platform vs. TELECARE Platform; (**e**). Respondents’ perception of the level of information given by patients to healthcare professionals provided on REACTS platform vs. TELECARE Platform; (**f**). Respondents’ perception of the patients’ consideration as a full member of care team provided on REACTS platform vs. TELECARE Platform; (**g**). Respondents’ perception of the level of empathy shown to patients provided on REACTS platform vs. TELECARE Platform.

Lastly, the majority of them indicated that, overall, both the quality (Figure 1d) and safety (Figure 1e) of care provided had not changed (Figure 1). Overall, the participants in the various health care professional groups reported an almost similar perception of the added value of REACTS-Teleconsultation and Telecare-Covid (Figure 1 and Figure 2).

### 3.3. Perceptions of Patient Engagement in Care and the Relationship with the Care Team

Figure 2 shows the perceptions of patient engagement in care and the relationship with the care team for the various groups. Most participants were positive about their experience during the COVID-19 pandemic and the support provided to facilitate confidence and trust with patients (Figure 2a); to engage patient in the decisions related to care (Figure 2c); to share a good amount of information with patients (Figure 2d); to let the patients provide information on the state of their health condition (Figure 2e); to show their empathy for the patient (Figure 2g); and to consider the patient as a full member of the care team (Figure 2f). In addition, the majority of participants in both groups indicated that they were able to help the patients become more autonomous (Figure 2b).

Among REACTS-Teleconsultation users, a higher proportion of individuals in “other” professional groups believed that they could give patients the means to help them become more autonomous (Figure 2b). However, among the participants who evaluated the added value of Telecare-Covid, there was a non-significant trend toward a higher proportion of individuals in the “nurses” professional group who believed that they could give patients the means to help them become more autonomous.

In addition, a higher proportion of individuals in “other” professional groups ensured that patients were always able to provide information on the state of their health. There were no significant differences in perceptions among the various groups of health care professionals evaluating the two platforms with respect to creating a bond of confidence/trust, patient engagement in decision making, patient independence, and showing empathy for the patient (Figure 2).

### 3.4. Perceptions of the Role and Relevance (Usefulness, Advantages, and Limitations) of REACTS-Teleconsultation

Survey participants who used REACTS-Teleconsultation were asked to provide more detailed information on their experience with this method. Overall, the findings demonstrate remarkable levels of appreciation for the platform. Fully 60.6% of REACTS-Teleconsultation users indicated that the platform was a good response to their needs or their patients’ needs (useful) (slightly agree to completely agree). In addition, 70% of health care professionals indicated that using the platform/technology reduced their daily use of personal protective equipment (PPE) (slightly agree to completely agree). In addition, 46.2% of all participants suggested that use of REACTS-Teleconsultation should be maintained after the health crisis (Table 3).

The participants in the various health care professional groups reported different experiences using REACTS-Teleconsultation, with a higher satisfaction rate among non-physician/non-nurse health care professionals compared with physicians and the “other” group (Table 3).

The most frequently reported problem encountered with REACTS-Teleconsultation concerned technical difficulties caused by being at a distance for certain tasks/exams: 56.4% (Table 4). There were no significant differences in perceptions of the limitations of REACTS-Teleconsultation among the various groups of health care professionals (Table 4).

### 3.5. Perceptions of the Role and Relevance (Usefulness, Advantages, and Limitations) of Telecare-Covid

Survey participants who evaluated the added value of Telecare-Covid were also asked to provide more detailed information on their experiences with this method. Overall, the findings demonstrated remarkable levels of appreciation for the platform. Fully 85.7% of participants indicated and completely agreed that the platform was useful and was a good response to their needs or their patients’ needs (Table 3). In addition, 64.3% of them suggested that use of Telecare-Covid should be maintained after the health crisis (Table 3).

The participants in the various health care professional groups reported different perceptions of Telecare-Covid (Table 3). The most frequently reported limitation was the lack of training and/or direct support, reported by 25% of the participants who evaluated Telecare-Covid. No significant differences were found among the participants in the various health care professional groups concerning their perceptions of the limitations of Telecare-Covid (Table 4).

## 4. Discussion

The novel coronavirus disease (COVID-19) has brought unprecedented changes to how conventional health care is delivered. Hospitals had to adjust the way they provide health care and become testing grounds for innovations to minimize the impact of patient surges on facilities. Many telehealth technologies, such as Telecare-Covid, were multidisciplinary virtual platforms that existed long before the pandemic and were further developed and adapted within a limited time frame for the remote monitoring of COVID-19 patients, inside and outside a healthcare organization.

Overall, our study findings are encouraging, and the dimensions evaluated demonstrate remarkable levels of appreciation for both Telecare-Covid and REACTS-Teleconsultation. The survey results showed that, in general, healthcare professionals’ perceptions of the quality and safety of care provided on the two remote monitoring platforms were positive, suggesting that these platforms have helped maintain a satisfactory level of quality and safety of care in the continuum of care.

For both platforms, we identified two main features that were highly appreciated by the majority of the professionals: they mainly liked the ease of access to their services in general, and they especially appreciated the reduced waiting times for patients. Although there were indications of some problems with these two platforms, such as a lack of training and/or direct support, about half of the healthcare professionals suggested that these platforms should be maintained after the health crisis.

Similar to our findings, in the literature, we found several previous studies suggesting that telehealth platforms have positive impacts, particularly on the quality and safety of care [6,9,10,11,12,13,14]. Positive impacts have also been demonstrated on the acceptability, usefulness, and user-friendliness of the technological tools and devices used in telehealth platforms in several clinical fields, notably in long-term care, mental health, oncology, etc.

More recently, telehealth platforms have been extensively studied, tested, and demonstrated in the clinical context of COVID-19 [29,30,31,32,33,34]. However, these studies mainly assessed patients’ experience with platforms, while less attention has been paid to various health care professionals’ perspectives on the usefulness of these platforms [29,30,31,32,33,34]. In addition, our study is the first to report on REACTS-Teleconsultation, which is a collaborative telehealth platform for remote meetings between patients and health professionals.

It is interesting to note that the participants in the various health care professional groups reported slightly different experiences resulting from their use of REACTS-Teleconsultation and Telecare-COVID, especially in terms of the obstacles faced or problems encountered in routine use. A higher proportion of non-physician/non-nurse health care professionals complained about the lack of training or direct support for their use of REACTS-Teleconsultation, a certain dehumanization of the relationship with the patient, and certain technical difficulties caused by being at a distance for certain tasks and exams compared with the “other” group and physicians.

Several studies have identified training on how to use digital health platforms as one of the major determinants of positive opinions and higher acceptability among health professionals [35,36,37,38,39,40]. These studies considered that health professionals who are well trained on how to use a digital platform feel well equipped, confident, and comfortable in their use of the technology [35,36,37,38,39,40].

Furthermore, these studies suggest that health professionals who are well trained on digital platforms trust technological platforms more and show less fear of information leaks and confidentiality issues, but also that they do not consider the care they provide through these platforms as dehumanized [35,36,37,38,39,40]. The findings of these studies may be taken as a clear indication that training is key when implementing digital health platforms and promoting their use among health professionals.

In line with our findings, previous studies have shown that views on healthcare technologies are linked to views on professional status, and that it was mainly the non-physician/non-nurse health care professionals who were concerned about potential impacts on the stability of existing patient–professional relationships [41,42].

These differing viewpoints may be related to levels of experience with the platforms and training on them. In our study, the non-physician/non-nurse health care professionals were mainly rehabilitation professionals, such as speech and respiratory therapists, physiotherapists, kinesiologists, etc. Again, these results agree with recent studies indicating that rehabilitation professionals, such as physiotherapists and speech and language therapists, may benefit from using Telehealth [41,42,43,44].

Furthermore, nurses reported an overall positive perception of the test platforms in our study. For this group of professionals, online platforms can be used to provide services, such as consultative care, triage assistance, and support for the clinician with the patient [44,45]. In addition, previous studies have shown that nurses usually welcome online platforms for patient care [13,44,45,46,47]. However, several barriers may be encountered when nursing services are provided through these platforms. Examples of the barriers reported by nurses in this study include lack of training and dehumanization of the relationship with the patient. Therefore, guidelines need to be developed for telehealth nursing care.

Another interesting finding is that the majority of individuals who indicated that they had no problems using REACTS-Teleconsultation were in the “other” group. This result is interesting but not surprising, since we believe that the interventions by health professionals in this category—in particular psychotherapists and social workers—are mostly conversational, where physical examination or interaction is not always required or necessary clinically. Therefore, telemedicine could be better suited to disciplines that do not require a physical examination and interaction [44,47].

However, this group did not report a similar experience using Telecare-Covid. This group mainly consisted of volunteers and social service, spiritual healing, and psychology professionals. This suggests that REACTS-Teleconsultation has considerable potential as a way to assist psychologists and social workers and increase the reach of mental health services during the COVID-19 pandemic but also for continued use and development after the pandemic. The usefulness of telepsychology has previously been demonstrated in several pre-and post-pandemic studies, from the perspectives of not only health professionals but also patients and the providers and users of mental health services [48,49,50,51].

We are planning another study that will explore and assess this dimension from patients’ perspectives, to see whether the two platforms will be also well received by patients and how they will rate them, and whether they see them as an acceptable means for mental health service delivery [52]. However, our results from all the categories of health professionals in our study suggest that there are some indications of uncertainty and fear of information leaks and over the lack of confidentiality on REACTS-Teleconsultation. These concerns need to be addressed to fully engage health professionals in adopting this digital platform [53,54,55].

Currently, policymakers are evaluating whether to maintain many of the policy changes implemented for the public health emergency [56,57]. Our results suggest that ongoing use of REACTS-Teleconsultation or Telecare-Covid may help maintain care and increase convenience for both patients and many health care professionals.

However, some improvements are needed regarding the technical and practical aspects of the platforms: (1) formally training health care professionals on how to use the platforms, technically and practically, to promote their adoption and use; (2) developing and enhancing the correspondence mechanism to speed up the communication and exchange process between health professionals and between patients and care teams and make it more responsive; and (3) developing and adapting the platforms’ content to the needs of COVID-19 patients with chronic diseases and adding more clinical profiles to the platforms to provide a more specific, more customized, and less generic follow-up process.

Finally, it should be noted, as mentioned above, that the platforms were not initially designed to monitor COVID-19 patients; rather, they were multidisciplinary virtual platforms that existed long before the pandemic. However, in order to quickly respond to the need to intervene and support care services and health professionals and maintain continuous, safe care of good quality, even if the care is provided remotely, it was decided to develop and adapt the existing platforms, within a short timeframe, for the remote monitoring of COVID-19 patients.

Therefore, in addition to the encouraging results that we have presented, we would like to highlight the success of the decision-making and technical transformation process that allowed us to better exploit the two platforms and quickly respond to urgent needs. This paper provides a sense of the effective collaboration achieved between the REACTS-Teleconsultation and Telecare-Covid technical teams and the leaders of CHUM and CRCHUM, and the considerable effort invested in this program, which could be considered a good model.

The current study has certain advantages. Several stakeholders, researchers, and experts in the field either supervised or were involved in the study. Our intervention was rigorously developed. There is also the original nature of this study, as the program of remote monitoring platforms and their evaluation was quickly planned and implemented, early in the first wave of the COVID-19 pandemic. It is worth mentioning that the participants in this study were also asked to evaluate other programs and/or platforms developed during the first wave of COVID-19. However, the present paper only presents results on Telecare-Covid and REACTS-Teleconsultation and focuses solely on their evaluation.

However, our study has several limitations. First, it was a single-center study with a relatively small sample size, especially for Telecare-Covid users. Second, the lack of a control group, and the fact that many of the participants who used these two technologies also used one or more other methods (multiple options were allowed) made comparisons between the platforms impossible. Therefore, this study did not examine the differences in user experience between the two platforms. Third, this study provides data from the first few months of the pandemic, and user experiences are likely to change over time.

Our study was not designed to assess whether changes in the services delivered by health care professionals through the platforms had an impact on patient satisfaction and outcomes. Lastly, another limitation that is beyond our control may be the low response rate. We believe that a higher response rate with more participation by healthcare professionals would definitely have strengthened our findings and deepened our understanding and conclusions of the challenge and trends related to the utilization of RPM by healthcare professionals.

## 5. Conclusions

Overall, the feedback and participants’ views we explored were positive. This study provides preliminary evidence suggesting that the two remote monitoring platforms we evaluated, REACTS-Teleconsultation and Telecare-Covid, were perceived as useful and user-friendly and were well-received by users, suggesting that they can be considered for use even in a post-pandemic era. However, our study also highlighted the fact that platform experience and awareness levels were still low, especially among non-physician/non-nurse health care professionals.

Hence, formal training is needed for health care professionals on how to use the platforms. If we want to continue to use these telehealth and similar digital platforms, there are still significant barriers that need to be resolved, and training appears to be the key. This has been often raised in the literature highlighted in the present paper. Thus, to improve the two platforms and maximize their use, the areas for improvement and the issues identified should be addressed by taking a collaborative approach with both health professionals and patients and also involving health system leaders, decision makers, and digital platform providers.

Ultimately, training is one of the major aspects on which health systems should focus in order to promote the adoption and improve the use of health platform technologies. This study makes a modest contribution toward enriching and deepening the knowledge available in the literature in the field of telehealth and telemonitoring in general, and in particular to knowledge on the impacts and challenges of using such approaches in an extraordinary context.

## Figures and Tables

**Table 1:** Dimensions and items studied through the survey.

Section/Dimension	Questionnaire Item/Attribute
Section 1, Demographic characteristics	Gender
	Age
	Profession (physician, nurse, non-physician/non-nurse, other)
	Years of experience
Section 2, Perceptions of performance and of quality and safety of care	The work I did made sense to me.
	My work environment during the COVID-19 health crisis allowed me to perform at a good level in my work.
	Overall, I think the quality of care we provide has been… (select a response)
	Overall, I think the safety of care we provide has been… (select a response)
	The measures taken to reduce the risk of contamination were adequate.
Section 3, Perceptions of patient engagement in care and the relationship with the care team	I ensured that a bond of confidence/trust was created with patients.
	I gave patients the means to help them become more autonomous.
	I made sure that the patient was engaged in the process and participated in the decisions related to care.
	I was able to provide the same amount of information about care to the patients as I do in normal times.
	I ensured that patients were always able to provide me with information on the state of their health (their health condition).
	I made a point of showing empathy for the patient.
	I considered the patient as a full member of the care team.
Section 4. Perceptions of the role played by the technology/platform and its relevance (usefulness, advantages, and limitations)	The platform/technology is a good response to my needs or my patients’ needs (useful).
	What were the advantages of using the platform/technology?
	What obstacles or problems prevented routine use of the platform/technology?
	Using the platform/technology reduced my daily use of personal protective equipment (PPE).
	Indicate whether the measure(s) should be maintained after the health crisis.

**Table 2:** Characteristics of participants who evaluated REACTS-Teleconsultation and Telecare COVID.

Characteristics	Total (*n* = 491)	REACTS-Teleconsultation		*p*-Value	Telecare-Covid		*p*-Value
		Yes (*n* = 128)	No (*n* = 364)		Yes (*n* = 34)	No (*n* = 458)	
Gender *, *n* (%)							
Male	111 (22.7)	26 (20.3)	85 (23.5)	0.587	10 (29.4)	101 (22.2)	0.546
Female	377 (76.9)	101 (78.9)	276 (76.2)		24 (70.6)	353 (77.4)	
Age *, *n* (%)							
<24 years	30 (6.1)	2 (1.6)	28 (7.7)	0.001	3 (8.8)	27 (5.9)	0.364
25–34 years	157 (32.0)	39 (30.5)	118 (32.5)		6 (17.7)	151 (33.0)	
35–44 years	146 (29.7)	48 (37.5)	98 (27.0)		12 (35.3)	134 (29.3)	
45–54 years	112 (22.8)	35 (27.3)	77 (21.2)		11 (32.4)	101 (22.1)	
≥55 years	46 (9.4)	4 (3.1)	42 (11.6)		2 (5.9)	42 (9.6)	
Professions, *n* (%)							
Physicians	40 (8.2)	15 (11.8)	25 (6.9)	<0.001	16 (47.1)	24 (5.3)	<0.001
Non-physician/non-nurse health care professionals	294 (59.9)	85 (66.9)	209 (57.4)		10 (29.4)	284 (62.1)	
Nurses	81 (16.5)	8 (6.3)	73 (20.1)		5 (14.7)	76 (16.6)	
Other	76 (15.5)	19 (15.0)	57 (15.7)		3 (8.8)	73 (16.0)	
Years worked **							
Median (IQR)	9 (13.0)	10 (13.3)	8 (13.0)	0.563	10 (13.0)	9 (13.0)	0.735

* Two missing/unknown (0.5%) for gender. One missing for age (0.3%). ** Three missing for the number of years worked.

**Table 3:** Perceptions of participants who evaluated the role and relevance (usefulness, advantages, and limitations) of REACTS-Teleconsultation.

The Platform/Technology is a Good Response to My Needs or Patients’ Needs (Useful)	REACT-Teleconsultation *n* = 127 *n* (%)						Telecare-Covid *n* = 28 *n* (%)					
	All	Physicians	Non-Physician/Non-Nurse Health Care Professionals	Nurses	Others	*p*-Value	All	Physicians	Non-Physician/Non-Nurse Health Care Professionals	Nurses	Others	*p*-Value
Completely disagree	5 (100)	3 (60.0)	1 (20.0)	0 (0.0)	1 (20.0)	NA	0 (0.0)	0 (0.0)	0 (0.0)	0 (0.0)	0 (0.0)	0.983
Slightly disagree	17 (100)	4 (23.5)	12 (70.6)	1 (5.9)	0 (0.0)		3 (100)	2 (12)	1 (10)	0 (0.0)	0 (0.0)	
Neutral	12 (100)	2 (16.7)	5 (41.7)	4 (33.3)	1 (8.3)		3 (100)	2 (66.7)	1 (33.3)	0 (0.0)	0 (0.0)	
Slightly agree	39 (100)	1 (2.6)	33 (84.6)	0	5 (12.8)		10 (100)	6 (60)	2 (20)	1 (10)	1 (10)	
Completely agree	38 (100)	5 (13.2)	22 (57.9)	2 (3.3)	9 (23.7)		14 (100)	5 (35.7)	4 (28.6)	3 (21.4)	2 (14.3)	
I do not want to answer/I do not know/Does not apply	16 (100)	0 (0.0)	12 (75.0)	1 (6.2)	3 (18.8)		4 (100)	1 (25.0)	2 (50.0)	1 (25.0)	0 (0.0)	
What were the advantages of using the platform/technology?												
Increased accessibility of services	43 (100)	11 (25.6)	18 (41.9)	NA	14 (32.6)	0.568	5 (100)	2 (40.0)	3 (60.0)	NA	0 (0.0)	0.054
Reduced waiting time	21 (100)	2 (9.5)	12 (57.1)	NA	7 (33.3)	0.028	4 (100)	1 (25.0)	2 (50.0)	NA	1 (25.0)	0.266
Improved quality of care	11 (100)	3 (27.3)	7 (63.6)	NA	1 (9.1)	0.086	5 (100)	2 (40.0)	3 (60.0)	NA	0 (0.0)	0.054
Improved efficiency of care	19 (100)	6 (31.6)	10 (52.6)	NA	3 (15.8)	0.093	3 (100)	1 (33.3)	1 (33.3)	NA	1 (33.3)	0.678
Increased number of times we can interact	20 (100)	4 (20.0)	10 (50.0)	NA	6 (30.0)	0.378	2 (100)	1 (50.0)	1 (50.0)	NA	0 (0.0)	0.522
Improved access and speed of care	25 (100)	6 (24.0)	12 (48.0)	NA	7 (28.0)	0.386	4 (100)	0 (0.0)	3 (75.0)	NA	1 (25.0)	0.011
Promotes user participation (user-partner approach)	21 (100)	4 (19.0)	14 (66.7)	NA	3 (14.3)	0.002	4 (100)	2 (50.0)	0 (0.0)	NA	2 (50.0)	0.244
Optimization of the use of resources (Adequate use and accessibility to skills)	17 (100)	4 (23.5)	9 (52.9)	NA	4 (23.5)	0.302	3 (100)	1 (33.3)	2 (66.7)	NA	0 (0.0)	0.119
Support for integrated service networks (inter-professional collaboration and service integration)	8 (100)	0 (0.0)	6 (75.0)	NA	2 (25.0)	0.047	2 (100)	0 (0.0)	1 (50.0)	NA	1 (50.0)	0.238
Using the platform/technology reduced my daily use of personal protective equipment (PPE)												
Completely disagree	8 (100)	1 (12.5)	5 (62.5)	1 (12.5)	1 (12.5)		10 (100)	4 (40)	2 (20)	2 (20)	2 (20)	
Slightly disagree	9 (100)	2 (22)	5 (55.6)	1 (11.1)	1 (11.1)		1 (100)	1 (100)	0	0	0	
Neutral	9 (100)	1 (11.1)	4 (44.5)	2 (22.2)	2 (22.2)	0.149	7 (100)	6 (85.7)	0	1 (14.3)	0	0.318
Slightly agree	29 (100)	3 (10.3)	20 (69.0)	2 (6.9)	4 (13.8)		7(100)	2 (28.6)	4 (57.1)	1 (14.3)	0	
Completely agree	55 (100)	8 (14.5)	37 (67.3)	0	10 (18.2)		4 (100)	2 (50.0)	1 (25.0)	0	1 (25.0)	
I do not want to answer/I do not know/Does not apply	17 (100)	0	14 (82.3)	2 (11.8)	1 (5.9)		5 (100)	1 (20.0)	3 (60.0)	1 (20.0)	0	
Indicate whether the measure(s) should be maintained after the health crisis	54 (100)	14 (25.9)	26 (30)	NA	14 (25.9)	0.003	18 (100)	9 (50.0)	5 (27.8)	NA	4 (22.2)	0.493

Abbreviations: NA, not applicable.

**Table 4:** Perceptions of participants who evaluated the role and relevance (usefulness, advantages, and limitations) of Telecare-Covid.

Problems/Difficulties Encountered	REACT-Teleconsultation *n* = 117 *n* (%)						Telecare-Covid *n* = 28 *n* (%)					
	All	Physicians	Non-Physician/Non-Nurse Health Care Professionals	Nurses	Other	*p*-Value	All	Physicians	Non-Physician/Non-Nurse Health Care Professionals	Nurses	Other	*p*-Value
Lack of training and/or direct support for use	34 (100)	5 (14.7)	24 (70.6)	1 (2.9)	4 (11.8)	0.722	10 (100)	7 (70.0)	0	1 (10.0)	2 (20.0)	0.534
Lack of usability of these technologies	39 (100)	9 (23.1)	24 (61.5)	1 (2.6)	5 (12.8)	0.068	3 (100)	2 (66.7)	0	0	1 (33.3)	0.608
Lack of interest in these technologies	14 (100)	3 (21.4)	8 (57.1)	1 (7.1)	2 (14.3)	0.600	3 (100)	1 (33.3)	1 (33.3)	1 (33.3)	0	0.522
Additional workload	43 (100)	8 (18.6)	28 (65.1)	1 (2.3)	6 (14.0)	0.273	6 (100)	2 (33.3)	2 (33.3)	1 (16.7)	1 (16.7)	0.393
Lack of time	21 (100)	5 (23.8)	13 (61.9)	1 (4.8)	2 (9.5)	0.312	8 (100)	4 (50.0)	3 (37.5)	1 (12.5)	0	0.801
Technical difficulties caused by being at a distance for certain tasks/exams	67 (100)	6 (9.0)	54 (80.0)	1 (1.5)	6 (9.0)	NA	5 (100)	3 (60.0)	2 (40.0)	0	0	0.525
Fear of a lack of confidentiality and of leaks of the information exchanged	15 (100)	3 (20.0)	5 (33.3)	0	7 (46.7)	NA	3 (100)	3 (100.00)	0	0	0	0.678
Dehumanization of the relationship with the patient	30 (100)	2 (6.7)	17 (56.7)	2 (6.67)	9 (30.0)	0.067	4 (100)	0	0	0	0	NA
Non-integration into our current technological systems and practices	13 (100)	2 (15.4)	6 (46.2)	0	5 (38.5)	0.066	6 (100)	1 (25.0)	1 (25.0)	1 (25.0)	1 (25.0)	0.608
Other problems	23 (100)	3 (13.0)	15 (65.2)	0	5 (21.7)	0.512	2 (100)	5 (83.3)	1 (16.7)	0	0	0.721
No problem encountered	12 (100)	1 (8.3)	6 (50.0)	1 (8.33)	4 (33.3)	0.225	2 (100)	0	1 (50.0)	1 (50.0)	0	0.678
I do not wish to answer/I do not know/Does not apply	7 (100)	0	3 (42.9)	4 (57.1)	0	NA	9 (100)	1 (50.0)	1 (50.0)	0	0	0.522

Abbreviations: NA, not applicable.

## Data Availability

The data presented in this study are available on request under the corresponding author.
